# Effect of Bladder Catheterization On Bacterial Interference With Asymptomatic *Escherichia coli* Strain 83972 in an Experimental Porcine Model of Urinary Tract Infection

**DOI:** 10.1093/infdis/jiae404

**Published:** 2024-08-20

**Authors:** Kristian Stærk, Karin Andersen, Janni Søvsø Hjelmager, Louise Kruse Jensen, Benjamin Meyer Jørgensen, Jakob Møller-Jensen, Lars Lund, Thomas Emil Andersen

**Affiliations:** Department of Clinical Research, University of Southern Denmark, Odense, Denmark; Department of Clinical Microbiology, Odense University Hospital, Odense, Denmark; Department of Clinical Research, University of Southern Denmark, Odense, Denmark; Department of Urology, Odense University Hospital, Odense, Denmark; Department of Clinical Research, University of Southern Denmark, Odense, Denmark; Department of Veterinary and Animal Sciences, University of Copenhagen, Copenhagen, Denmark; Department of Veterinary and Animal Sciences, University of Copenhagen, Copenhagen, Denmark; Department of Biochemistry and Molecular Biology, University of Southern Denmark, Odense, Denmark; Department of Clinical Research, University of Southern Denmark, Odense, Denmark; Department of Urology, Odense University Hospital, Odense, Denmark; Department of Clinical Research, University of Southern Denmark, Odense, Denmark; Department of Clinical Microbiology, Odense University Hospital, Odense, Denmark

**Keywords:** urinary tract infection, *E. coli* ABU 83972, asymptomatic bacteriuria strain, bladder catheter, porcine model

## Abstract

**Background:**

Urinary tract infection (UTI) is a common disease with a significant risk of relapse. Deliberate bladder colonization with asymptomatic *Escherichia coli* is being explored as a potential strategy to fend off invading uropathogens, thereby mitigating the risk of symptomatic UTI. Currently, one major obstacle is the low success rates for achieving persistent bladder colonization with asymptomatic bacteria and experimental challenge studies are lacking. Here, we assessed the influence of an indwelling bladder catheter on the ability of asymptomatic *E. coli* to colonize the bladder and to assess the protective efficacy of such colonization against experimental UTI with uropathogenic *E. coli*.

**Methods:**

Pigs with or without indwelling bladder catheters were experimentally inoculated with the asymptomatic *E. coli* strain 83972 and subsequently challenged by inoculation with a uropathogenic *E. coli* isolate, UTI89. The animals were monitored with regular urine and blood samples and bladders and kidneys were harvested at termination.

**Results:**

All pigs with indwelling catheters were colonized by *E. coli* 83972 in response to inoculation, compared to pigs without catheters in which only 1 of 8 animals were colonized. When removing the catheter, *E. coli* 83972 were spontaneously cleared. Colonization with *E. coli* 83972 prevented experimental infection in 50% of animals, whereas all control animals became infected.

**Conclusions:**

The presence of indwelling bladder catheters strongly facilitates the colonization of *E. coli* 83972, indicating that individuals with catheters may be particularly suited for receiving this treatment. The research supports prophylactic colonization with *E. coli* 83972 as a potential strategy to reduce the risk of UTIs.

## Teaser

The presence of an indwelling urinary catheter significantly increases bladder colonization with the asymptomatic *Escherichia coli* bacteriuria strain 83972. Colonization with *E. coli* 83972 prevents 50% of experimental urinary tract infections.

Urinary tract infection (UTI) is among the most common bacterial infections and affects 150 million people each year worldwide [1]. Recurrent UTIs (rUTIs) are defined as simple or complicated UTIs occurring ≥3 times a year or twice within 6 months [2]. The concerning rise in antibiotic-resistant uropathogens presents significant challenges for individuals, leading to prolonged or failed treatment [3]. Antimicrobial resistance is predicted to be the cause of 10 million deaths worldwide in 2050, and *Escherichia coli* is identified as the most important contributor to antimicrobial resistance-related deaths [4, 5].

The most common causative agent of rUTI is uropathogenic *E. coli* (UPEC). The UPEC pathogenesis has been extensively studied over the last 2 decades in an attempt to clarify the underlying mechanism responsible for the high prevalence of rUTI [6]. No significant interventions have been introduced that effectively reduce the incidence of rUTI, and antibiotic consumption in this patient group remains high [5].

Bacterial interference refers to the antagonism between bacterial species during the process of niche colonization and acquisition of nutrients. The most studied asymptomatic bacteriuria (ABU) strain, *E. coli* 83972, is well adapted to growth in human urine, and it outcompetes UPEC during growth in vitro [7]. Clinical trials have shown that inoculation of bladders in rUTI patients with *E. coli* 83972 can result in asymptomatic bladder colonization which lowers the incidence of rUTI [8–12]. However, one major obstacle highlighted in most clinical studies is the low rate of success for achieving persistent bladder colonization with *E. coli* 83972 [13, 14].

Implanted or indwelling medical devices have long been known to promote bacterial colonization in the body and thus contribute to hospital-associated infections [15]. For deliberately promoting the bacterial colonization of specific sites in the body, however, foreign materials may prove advantageous, and approaches have therefore been made to design materials that contain and seed *E. coli* 83972 to interfere with UPEC growth [16]. Many of the patients who may benefit from a nonantibiotic prophylactic treatment against UTIs already carry implanted or indwelling devices [17]. We hypothesized that indwelling bladder catheters may also promote bladder colonization by *E. coli* 83972. In the current study, we sought to investigate the influence of an indwelling bladder catheter on the ability of *E. coli* 83972 to colonize the bladder and whether such colonization could protect against infection with a pathogenic strain.

We used an experimental infection model in pigs since the size of this animal supports human characteristics [18, 19]. Furthermore, UPEC is a zoonotic pathogen causing UTI in both pigs and humans, making pigs uniquely suited as animal model of UTI [20].

## MATERIALS AND METHODS

### Animals

Female domestic pigs (n = 22), 13–14 weeks of age, were sourced from a local supplier (Kokkenborg ApS, Stenstrup, Denmark) and allowed 7 days acclimatization before the beginning of experiments. Animals were housed in standard enclosures with a concrete floor and sawdust bedding. They were housed individually when they had an indwelling catheter and otherwise in communal enclosures with 3 m^2^ per animal. Enrichment was provided in the form of various toys, music, and daily human interactions. The study was approved by the Danish Animal Experiments Inspectorate (license no. 2021-15-0201-00931). Due to the large size of pigs, the study was performed in small groups of 4–6 animals per round and repeated. Since this study is the first of its kind to assess ABU colonization in pigs, this approach also facilitated intermittent evaluation of the outcomes between studies, to ensure that only an appropriate number of animals were used. A power calculation was performed before the study: with estimated colonization rates in catheterized and noncatheterized pigs of 70% and 10%, respectively, and a significance level of 5%, power of 80%, and attrition rate of 10%, 20 animals was determined to be the appropriate total number. The estimated colonization rates were based on existing literature and previous experience with the model.

### Bacteria

For asymptomatic colonization we used the reference ABU *E. coli* strain 83972. For infection challenge we used a human cystitis *E. coli* strain, UTI89. To facilitate differentiation between the 2 strains in biological samples, streptomycin resistance was induced in the *E. coli* 83972, and a UTI89Δ*LacZ* mutant with chloramphenicol resistance was constructed (Supplementary materials). To differentiate between the strains, we used agar plates supplemented with streptomycin or chloramphenicol as well as blue agar plates (SSI Diagnostica) indicative of lactose fermentation and Chromid CPS Elite agar plates (Biomérieux). The UTI89Δ*LacZ* grows as white colonies on blue agar, whereas *E. coli* 83972, being lactose fermenting, forms yellow colonies on this substrate (Supplementary Figure 1). On Chromid CPS Elite plates, *E. coli* 83972 grows as dark red colonies, and UTI89Δ*LacZ* as light red colonies. Relevant growth assays were performed to ensure that the mutants were not growth retarded compared with their wild-type counterpart (Supplementary Figure 2).

To prepare *E. coli* 83972 suspensions for inoculation, the bacteria was incubated overnight in 40 mL of lysogeny broth supplemented with streptomycin, after which 100 µL of culture was reinoculated overnight in 40 mL of lysogeny broth. The culture was then centrifuged for 20 minutes at 5000*g*, resuspended in 10 mL of 0.9% saline solution, and adjusted to an optical density at 600 nm of 1.0 (±0.03), yielding approximately 1 × 10^9^ colony-forming units (CFUs)/mL. From here, 1 mL was transferred to 100 mL saline solution, for a final inoculum of 1 × 10^7^ CFUs/mL. Aliquots were plated to verify inoculum.

UTI89Δ*LacZ* was prepared similarly with 2 exceptions: (1) broths were supplemented with chloramphenicol, and (2) the suspension was diluted to a final inoculum of 10^5^ CFUs/mL (verified by plating).

### Anesthesia

Pigs were premedicated with an intramuscular injection of medetomidine (0.05 mg/kg; Cepetor Vet), butorphanol (0.2 mg/kg; Butomidor Vet), and midazolam (0.2 mg/kg; Midazolam Hameln). When complete muscle relaxation was reached, the animals were moved to the operating bed, and anesthesia was induced and maintained with propofol.

### Pig Challenge

**Figure 1.**
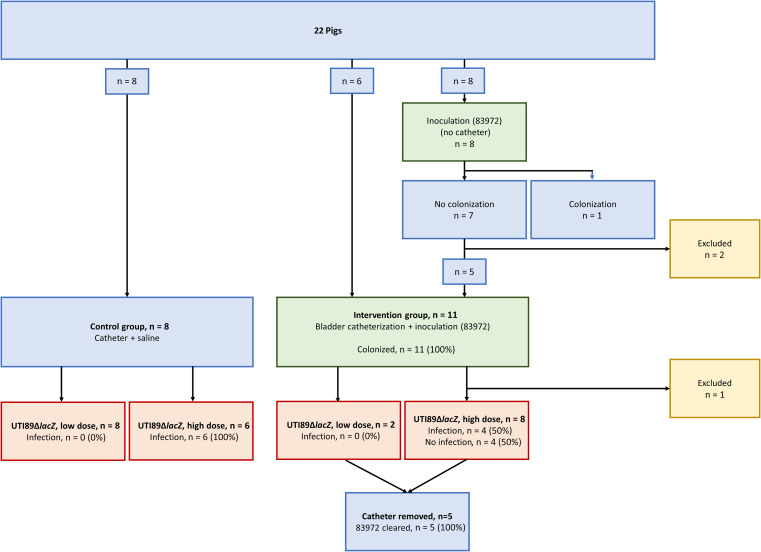
Group allocation of animals. Green boxes represent the procedure of inoculation with *Escherichia coli* strain 83972; red boxes, inoculation with UTI89Δ*lacZ*.

The experimental procedures and group allocations is summarized in a flowchart (Figure 1).

### Inoculation With *E. coli* 83972

Pigs were inoculated as described by Stærk et al [19]. Pigs were placed in dorsal recumbency, and the urogenital area was washed and disinfected. A 12F Foley-type catheter (Rüsch) was used to catheterize the animals and to allow instillation of an 100-mL inoculum. The catheter was clamped with a pean clamp for 1 hour to detain the inoculum, and after that the bladder was completely emptied to ensure equal basis of infection.

###  

For the experiment with indwelling catheters (intervention group), pigs were inoculated with *E. coli* 83972, in a procedure similar to that described above, but they were returned to their stables without removal of the catheter or emptying of the bladder. Catheters were equipped with a catheter valve that was changed daily to minimize contamination of the catheter, as previously demonstrated [18]. After 24 hours, the animals with indwelling catheters were reinoculated with a 30-mL inoculum (10^5^ CFUs/mL) preceded by 1-hour void restriction, after which the catheter valve was opened but the bladder was not manually emptied. Only 30 mL was used for reinoculation, as higher volumes (eg, 100 mL) are more likely to induce constraining in the animals (own observations). Control animals were mock-treated with saline solution. Indwelling catheters were always removed after 4 days of dwell time.

### Challenge With UTI89Δl*acZ*

Animals were challenged with UTI89Δl*acZ* 3 days after inoculation with *E. coli* 83972 (or mock treatment). The catheter valve was closed for 2 hours, after which the bladder was emptied and urine collected. After that, 30 mL of urine was mixed with 10 mL of UTI89Δ*LacZ* inoculum and instilled into the bladder through the catheter. The catheter was then flushed with 10 mL of saline solution and clamped for 1 hour. The catheters were then opened but not manually emptied. This approach was used to ensure equal basis of infection, so that varying volumes of bladder urine would not dilute the inoculum.

### Urine Collection

Baseline urine samples were collected immediately after catheterization. During follow-up with indwelling catheters, urine was collected by closing the catheter valve for 2 hours to allow accumulation of urine and then collecting the midportion of the urine directly from the catheter. All urines were cultured by plating 100-µL aliquots in serial dilutions and incubated overnight.

### Termination and Tissue Analysis

On the day of termination, urine was collected, and the catheters removed and processed as described below. The animals were then euthanized with 5 mL of intravenous pentobarbital (200 mg/mL), and their bladders removed aseptically post mortem. Bladders were opened by a midline incision between the ureters and washed by rinsing with saline solution. Seven tissue specimens were punched using a die (Ø = 10 mm), of which 2 were stored for histological analysis (see below) and 5 used for enumeration of adherent bacteria by removing the muscle layer and sonicating the remaining urothelium from all specimens in 4 mL of saline solution. After that, 100-µL aliquots were plated as described above.

### Catheter Analysis

10 mm pieces of tips and shafts were aseptically recovered from the catheters and sonicated for 5 minutes in 1 mL of saline solution to dissolve bacterial biofilm. Aliquots were plated as described above.

### Excluded Animals

One animal was euthanized on day 2 due to a false passage, and 4 animals (2 controls and 2 from the intervention group) were excluded because they were inoculated with a smaller inoculum (10^3^ CFUs/mL) not resulting in infection among the controls. A higher inoculum of 10^5^ CFUs/mL was chosen for the rest of the experiment. Two animals were excluded from the challenge experiment since their catheters had to be removed shortly after inoculation due to clinical signs of discomfort.

### Histology

Bladder specimens were fixed in 10% neutral buffered formalin (Sigma-Aldrich). After fixation, each specimen was cut into 2 parts. Both halves were placed in cassettes with the new sagittal cutting surface downward, processed through graded concentrations of alcohol, and embedded in paraffin wax. Tissue sections were cut (4 µm) and stained with hematoxylin-eosin (VWR, DK); stained sections were evaluated pathomorphologically with special focus on identifying tissue destruction and inflammation within mucosa and submucosa. The following parameters were registered with a yes or no: epithelial loss, epithelial ballooning, hemorrhage, hyperemia, edema, and neutrophil infiltration. Registration with a “yes” resulted in 1 point, and therefore a total of 6 points could be given for the histology score. Immunohistochemical staining based on antibodies to calprotectin was used to identify neutrophils; the protocol used has been published elsewhere [21].

### Statistical Analyses

Statistical analyses were performed using GraphPad Prism software (version 9.3.1). Comparisons between >2 groups of nonparametric data were performed using Kruskal-Wallis tests with Dunn multiple comparison tests. Contingency analysis was performed using Fisher exact tests. Differences were considered statistically significant at *P* < .05.

## RESULTS

### ABU Colonization and Infection Challenge

**Figure 2.**
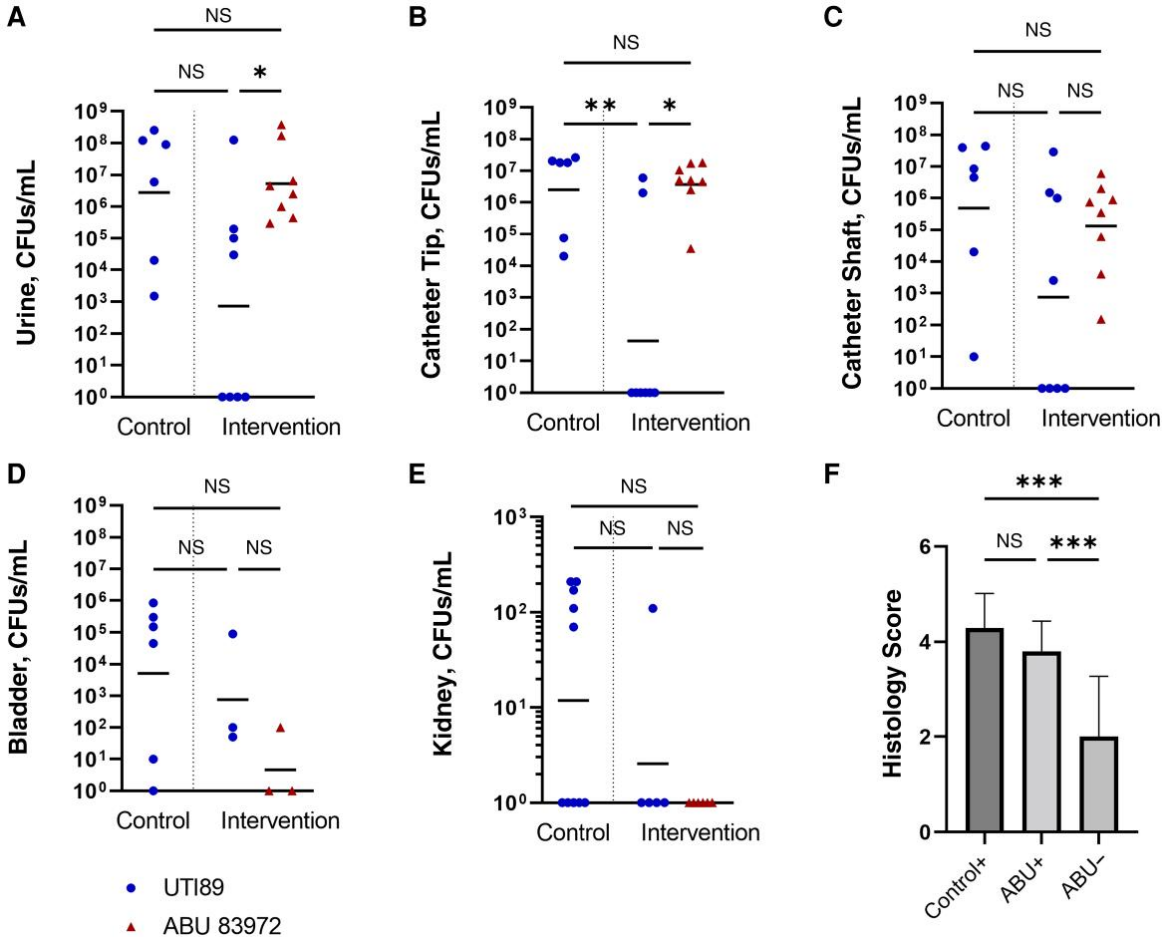
Challenge with UTI89Δ*lacZ* did not outcompete *Escherichia coli* strain 83972 in any of the pigs (n = 8). *A,* The bacterial burden of UTI89Δ*lacZ* in urine did not differ significantly between control and intervention groups, and in the intervention group UTI89Δ*lacZ* was significantly lower than *E. coli* 83972. *B,* In the intervention group, UTI89Δ*lacZ* was significantly lower on catheter tips compared with controls and also lower than *E. coli* 83972. *C,* On the catheter shaft, both strains were present in similar numbers. *D, E,* Strain 83972 was isolated from the bladder of only 1 pig (*D*) and never from the kidneys (*E*). Nonparametric Kruskal-Wallis tests were performed with Dunn multiple comparison tests (*A–E*). *F,* Pigs in the intervention group (ie, colonized with strain 83972) that resisted experimental infection with UTI89Δ*lacZ* (ABU−) had significantly less bladder inflammation than controls infected with UTI89Δ*lac* (Control+) and infected pigs in the intervention group (ABU+) (both *P* < .001). One-way analysis of variance was performed using Tukey multiple comparison test. Horizontal lines represent geometric mean. *P < .05; **P < .01; ***P < .001. Abbreviations: ABU, asymptomatic bacteriuria; CFUs, colony-forming units; NS, not significant.

Eight pigs were inoculated with *E. coli* 83972, with only 1 pig becoming colonized. At termination, this colonized animal showed hydroureter and hydronephroses suggestive of urinary tract abnormality. In comparison, all pigs with an indwelling urinary catheter (n = 11) became colonized (*P* < .001) (Table 1). Pigs with indwelling catheters and ABU colonization (n = 8) were subsequently challenged with high-dose UTI89Δ*LacZ*, resulting in 4 pigs (50%) with detectable UTI89ΔlacZ in urine 24 hours after inoculation, considered as manifest UTI (Table 1). UTI89Δ*LacZ* was undetectable in any biological sample from the other 4 animals. Challenge with UTI89Δ*LacZ* did not outcompete *E. coli* 83972 in any of the 4 animals with detectable UTI89Δ*LacZ* at 24 hours after inoculation (Figure 2*A*). All control animals (mock-colonized with saline solution) became infected with UTI89Δ*LacZ* in response to experimental inoculation with UTI89Δ*LacZ* (n = 6; *P* = .08) (Table 1).

**Table 1.** Inoculation outcomes

Colonization or Infection Challenge	Group	No.	Successful Colonization or Infection, No. (%)	*P* Value
Colonization with ABU strain 83972	No catheter	8	1 (12.5)	<.001
	Catheter	11	11 (100)	
UTI challenge	Control	6	6 (100)	.08
	ABU	8	4 (50)	

Abbreviations: ABU, asymptomatic bacteriuria; UTI, urinary tract infection.

Urine bacterial burden of UTI89Δ*LacZ* in infected pigs did not differ significantly between the control and intervention groups (geometric mean, 2.8 × 10^6^ vs 7.2 × 10^2^ CFUs/mL, respectively) (Figure 2*A*). In the intervention group, *E. coli* 83972 was present in significantly higher numbers than UTI89Δ*LacZ* (geometric mean, 5.3 × 10^6^ vs 7.2 × 10^2^ CFUs/mL, respectively) (Figure 2*A*). On the catheter tips, the mean CFU count for UTI89Δ*LacZ* in the intervention group was significantly lower than that for *E. coli* 83972 (*P* = .04) and UTI89Δ*LacZ* in the control group (*P* = .008). The shafts of the indwelling catheters were colonized by *E. coli* 83972 and UTI89Δ*LacZ* in similar numbers in both groups (Figure 2*C*). *E. coli* 83972 associated with bladder tissue was detected in only 1 animal (of 3) and *E. coli* 83972 was never detected in the kidneys (n = 6) despite significant urine bacterial burdens (Figure 2*D*). In infected pigs, UTI89Δ*LacZ* were generally detected in higher numbers in the bladder (up to 10^6^) and sometimes in the kidney, but only in very low numbers (often only a few colonies bordering the detection limit) (Figure 2*E*).

In 5 pigs of the intervention group the catheter was removed, resulting in spontaneous clearing of the ABU strain (determined by urine sampling 6–7 days after removal of the catheter). One of the pigs in the intervention group was infected with UTI89Δ*LacZ*, but in contrast to *E. coli* 83972 the UTI89Δ*LacZ* persisted to the end of study, 7 days after catheter removal.

### Histology

**Figure 3.**
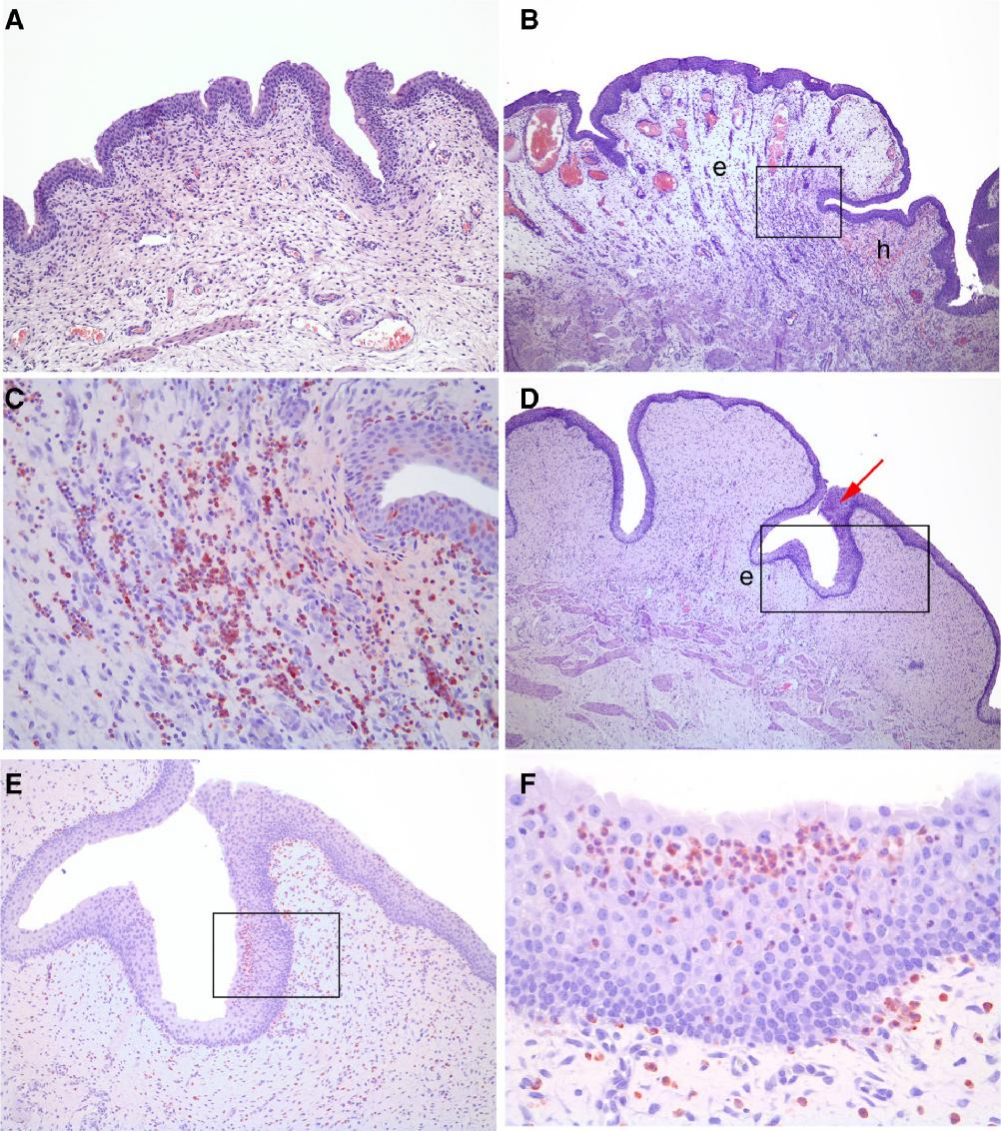
Histological findings in 3 study animals. *A,* No signs of inflammation are seen in pig 10-1, which cleared the infection (hematoxylin-eosin [HE], original magnification ×40). *B*, In pig 73, which became infected, edema (*e*), hyperemia, and hemorrhage (*h*) are seen in the lamina propia (HE, original magnification ×40). *C,* Close-up of inset in *B* shows massive infiltration of neutrophils in the lamina propia (immunohistochemical [IHC] stain, original magnification ×200). *D,* Edema (*e*) and epithelial hyperplasia (*arrow*) are seen in pig 76, a control pig with infection. (HE, original magnification ×40). *E,* Close-up of inset in *D* shows massive infiltration of neutrophils in both the epithelium and the lamina propia (IHC, original magnification 100). *F,* Close-up of inset in *E* (IHC stain, original magnification ×400). All IHC stains are based on antibodies to calprotectin, located in the cytosol of neutrophils.

The mean histology score (SD) in control pigs (4.3 [0.7]) did not differ significantly from that in the infected animals in the intervention group (3.8 [0.6]) (Figure 2). However, the pigs from the intervention group that resisted infection had a mean histology score (SD) (2.0 [1.2]) significantly lower than that in both controls pigs (*P* < .001) and infected pigs in the intervention group (*P* = .02). The inflammatory changes included loss of epithelium, hemorrhage, hyperemia, edema, neutrophil infiltration, macrophage infiltration, and epithelial ballooning (estimated to be a sublethal cell damage) (Figure 3). Hemorrhage and inflammatory cellular infiltration were seen within lamina propria and occasionally also inside the epithelium, respectively. The cellular infiltration was concentrated in foci. No inflammatory signs were seen in the bladder of pig 7, which had to be euthanized due to a false passage and therefore never had an indwelling catheter.

## DISCUSSION

The current study found that the ABU strain 83972 could not colonize the bladders of healthy pigs. In contrast, the presence of an indwelling catheter facilitated successful colonization in all animals. Catheter-promoted colonization with *E. coli* 83972 protected against experimental UTI in 50% of animals.

Spontaneous clearing of *E. coli* 83972 is a major obstacle for successful bacterial interference. Continuous colonization regimens with repetitive inoculations and use of strain 83972 mutants potentiated in adhesive pili have been some approaches to achieving long-term colonization, but even then the colonization rate remains low, warranting further investigations [13, 22].

In the current study we used, for the first time, the pig as a model animal for competition studies with an ABU strain. We found excellent colonization rates (100%) for pigs with an indwelling bladder catheter. The urine bacterial count of *E. coli* 83972 was significantly higher than that of UTI89Δ*LacZ* when both strains were present simultaneously. This is congruent with other studies in which *E. coli* 83972 was shown to outcompete pathogenic strains [7]. Significant colony counts (>10^5^ CFUs/mL) of strain 83972 were recovered from the catheter tip and shaft, demonstrating an excellent ability for this isolate to colonize a foreign body in the bladder. Bacterial attachment and colonization of the catheter surface is likely the main reason for achieving successful colonization in these animals. However, the presence of an indwelling catheter may predispose to a small amount of residual urine, in the bottom of the bladder around the balloon, that could be a contributing factor for bacterial persistence. The bacterial counts of UTI89Δ*LacZ* were significantly lower on the catheter tip from pigs in the intervention group, demonstrating that *E. coli* 83972 colonization also has the capacity to reduce catheter colonization by UPEC.

Continued challenges in maintaining colonization in the long term must be expected due to clearing of *E. coli* 83972 right after the indwelling catheter is removed. Likewise, none of the pigs with spontaneous voiding could keep a measurable colonization. Taken together, our findings suggest that uncompromised bladder function is an obstacle for effective colonization with strain 83972 and may explain why many studies have struggled with low colonization rates or focused only on patients with dysfunctional voiding. In the future, compromised bladder function may be a relevant criterion for the decision to attempt colonization with asymptomatic strains. A small study in patients with chronic indwelling catheters showed a high colonization rate (10 of 12 patients) using catheters coated with the strain 83972 derivative *E. coli* HU2117 (83972 with a *papG* deletion), and in another trial it was possible to colonize intermittent catheter users (colonization rates between 37% and 62%) suggesting that patients other than those with an indwelling catheter will be able to benefit from *E. coli* 83972 colonization in the long term [9, 12, 13, 23].

We identified a UTI protection rate of 50% in *E. coli* 83972–colonized pigs, congruent with findings in clinical studies of direct bladder inoculation with *E. coli* 83972 or HU2117 [8–11, 24]. This emphasizes the translatability of results from pigs to humans and suggests that colonization can be used in selected patients to avoid persistent and long-term antibiotic treatment. Horwitz and coworkers [25] showed a high colonization rate with HU2117-coated catheters among patients with chronic indwelling catheter (8 of 10 participants). Despite successful colonization, that study found that strain HU2117 did not effectively prevent bladder colonization by pathogenic bacteria or catheter-associated UTI. Although the study was small without a control group, its findings suggest that some patient groups (elderly patients in this case) may benefit less from interventional colonization with ABU. In the current study, pigs were experimentally inoculated with a pathogenic *E. coli* after 3 days of colonization with *E. coli* 83972. Prolonging the study period with the colonized indwelling catheter would likely have resulted in cocolonization of the catheter with other bacteria, including potential uropathogens, and we cannot rule out the possibility that this could eventually give rise to symptomatic UTI.

Bladder inflammation was found to be similar in infected pigs, independent of *E. coli* 83972 colonization. This suggests that while strain 83972 colonization may reduce susceptibility to infection, it does not reduce symptoms when an infection occurs.

The finding that ABU 83972 is unable to colonize healthy pig bladders sharply contrasts with findings of similar studies performed in murine models. Competition studies of *E. coli* 83972 against uropathogens have so far been performed only in mice, which can be consistently colonized with strain 83972 in the bladder and kidneys for at least 48 hours [26, 27]. While mice are a valuable animal model for elucidating various aspects of bacterial virulence, they deviate significantly from humans in several aspects, including the ability to produce highly concentrated urine, which represses bacterial growth [28, 29]. Surface-associated colonization is therefore likely the main growth mode for UPEC in the mouse bladder, unlike in larger mammals and humans, where planktonic growth in urine is pronounced. Rapid proliferation in human urine is the main competitive advantage of *E. coli* 83972, and the strain lacks key adhesins used by UPEC to adhere to the uroepithelium [8]. Therefore, using the mouse model to assess competitiveness of strain 83972 may underestimate the true potential of the strain. The use of pigs is a significant strength in the current study, and the findings of this study in terms of *E. coli* 83972 colonization outcome, UTI susceptibility, and intervention protection rate are in accordance with clinical observations.

We found that a low-dose bladder inoculation of UTI89Δ*LacZ* (10^3^ CFUs/mL) was inadequate to induce infection, despite the presence of an indwelling catheter. This contrast previous findings in noncatheterized pigs, where we have shown that <10^2^ CFUs/mL was able to establish robust infection in pigs. Compared with previous studies, in which the bacteria were suspended in saline solution, animals in the current study were inoculated with bacteria suspended in urine. Since urine contain a spectrum of immune components and proteins specifically inhibiting UPEC adhesion, such as antibodies, leukocytes, cytokines, and Tamm-Horsfall protein, this methodological detail is likely critical for the susceptibility to infection.

When tissue-associated bacteria were assessed, *E. coli* 83972 was detected in low numbers in the bladder tissue of only 1 animal, and never in the kidneys. These findings are congruent with the fact that strain 83972 lacks important adhesins, such as type 1 fimbriae and P fimbriae, preventing effective tissue colonization and dissemination to the kidneys. Taken together, the study findings demonstrate the avirulent nature of this strain.

Some degree of inflammation was observed in pig bladders from animals colonized with *E. coli* 83972, although significantly lower than with UTI89Δ*LacZ* infection. This could suggest that ABU colonization alone may give rise to low-level inflammation but is more likely to be a result of the indwelling catheter. Previous studies in pigs have shown that even short-term catheterization can inflict bladder mucosal trauma and inflammation [21], similar to the present findings. We did not include catheterized control pigs without ABU inoculation, so the impact of indwelling catheters on pathological score could not be assessed which is a limitation in the current study.

In conclusion, the ABU strain 83972 lacks the ability to establish itself in the bladders of healthy pigs, a characteristic also reported in healthy humans. However, when an indwelling catheter was present, it facilitated successful colonization in all animals. The catheter-promoted colonization of strain 83972 provided protection against experimental UTI in 50% of animals, confirming the bacterial interference potential of the strain. This 50% protection rate aligns with findings from prior clinical studies, implying that ABU colonization might be a viable strategy, particularly in patients with indwelling catheters, to circumvent the need for prolonged and persistent antibiotic treatment. The study findings demonstrate the appropriateness of pigs as animal models for ABU studies, since they uniquely reflect human susceptibility to asymptomatic and pathogenic strains, allowing for experimental infection studies.

## Supplementary Data

Supplementary materials are available at *The Journal of Infectious Diseases* online (http://jid.oxfordjournals.org/). Supplementary materials consist of data provided by the author that are published to benefit the reader. The posted materials are not copyedited. The contents of all supplementary data are the sole responsibility of the authors. Questions or messages regarding errors should be addressed to the author.

## Supplementary Material

jiae404_Supplementary_Data

## Notes

***Acknowledgments***. The authors thank the animal technicians and veterinarian Louise Langhorn from the biomedical laboratory at Odense University Hospital. Special thanks to Dr. rer. nat. Ulrich Dobrindt for kindly providing the ABU strain 83972.

***Financial support.*** The study was supported by the Region of Southern Denmark (grants R189-A4123-B2670 and R26-A1018 to K. A.), the Danish Association of Urology (grants to K. A.), and an IPSEN research legate (grants to K. A.).

***Data availability***. All data supporting the conclusions of this study are presented within the article and its supplementary materials.
