# Supplementary material for: Effect of Bladder Catheterization On Bacterial Interference With Asymptomatic Escherichia coli Strain 83972 in an Experimental Porcine Model of Urinary Tract Infection
Source: J Infect Dis. 2024 Aug 20;231(2):e355–63. doi: 10.1093/infdis/jiae404 (PMC12086672; doi:10.1093/infdis/jiae404)
Supplement: jiae404_Supplementary_Data [file jiae404_Supplementary_Data.zip › Supplementary_methods.docx]

# Supplementary Methods

**Construction of UTI89Δ*LacZ***

The E. coli UTI89 lacZ mutant was generated using the λ Red recombinase system as described by Datsenko and Wanner (2000) (https://www.pnas.org/doi/full/10.1073/pnas.120163297). A chloramphenicol resistance cassette was inserted to disrupt the lacZ gene.

Primer sequences used for the PCR amplification of the chloramphenicol cassette were:

JMJ1671 TCTTTACACTCTATGTGTCCGGCTCGTATGTTGTGTGAAATTGTGAGCGAATAACAATTTCACACAGGATGTGTAGGCTGGAGCTGCTTCG lacZ_KO_F

JMJ1672

AGTTTGTGTTTTTTAAATAGTACATAATGGATTTCCTTACGCGAAATACGGGCAGACATGGCCTGCCCGGCATATGAATATCCTCCTTAGTTCC lacZ_KO_R
