# Supplementary figures and images for: Effect of Bladder Catheterization On Bacterial Interference With Asymptomatic Escherichia coli Strain 83972 in an Experimental Porcine Model of Urinary Tract Infection
Source: J Infect Dis. 2024 Aug 20;231(2):e355–63. doi: 10.1093/infdis/jiae404 (PMC12086672; doi:10.1093/infdis/jiae404)

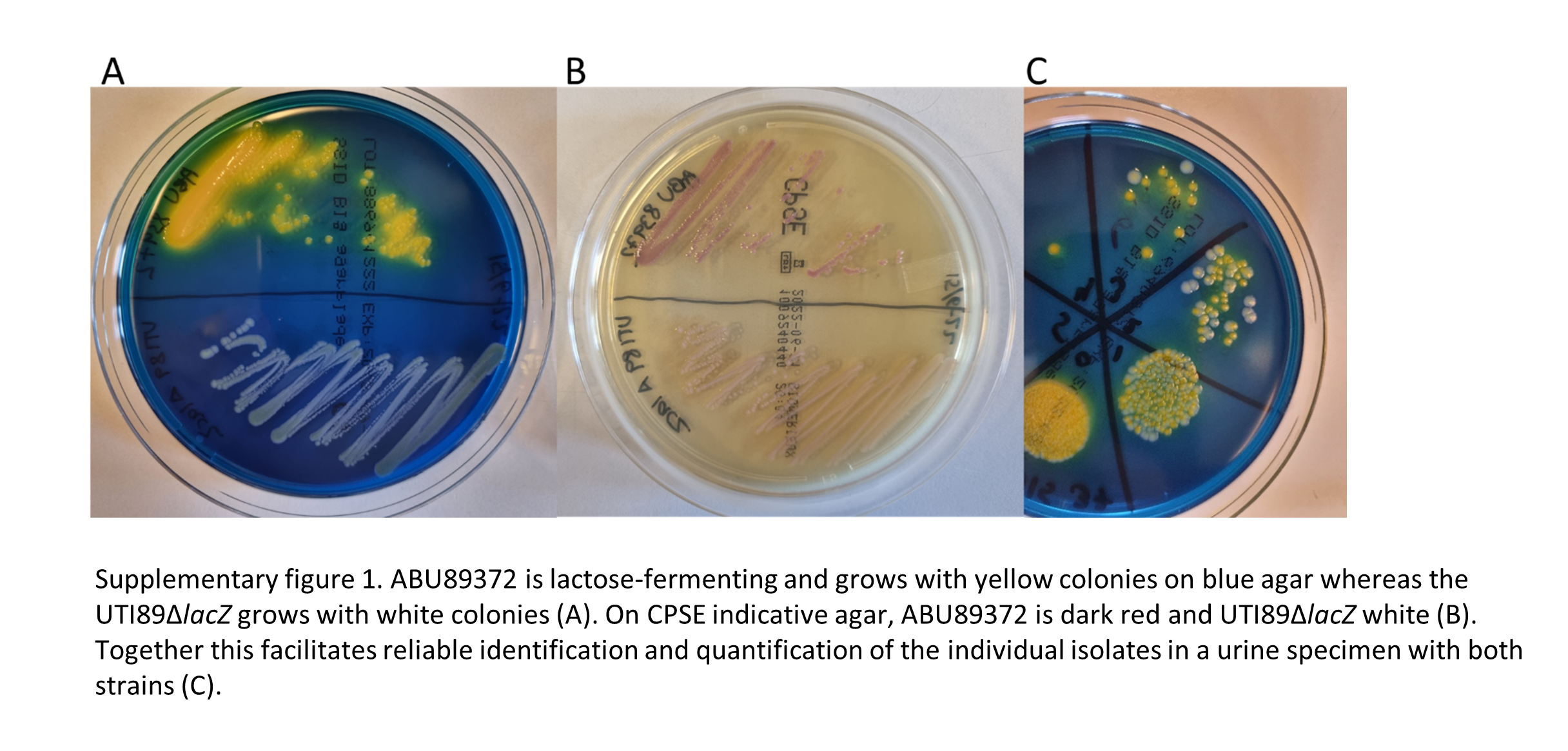

Supplement: jiae404_Supplementary_Data [file jiae404_Supplementary_Data.zip › Supplementary_figure1.png]

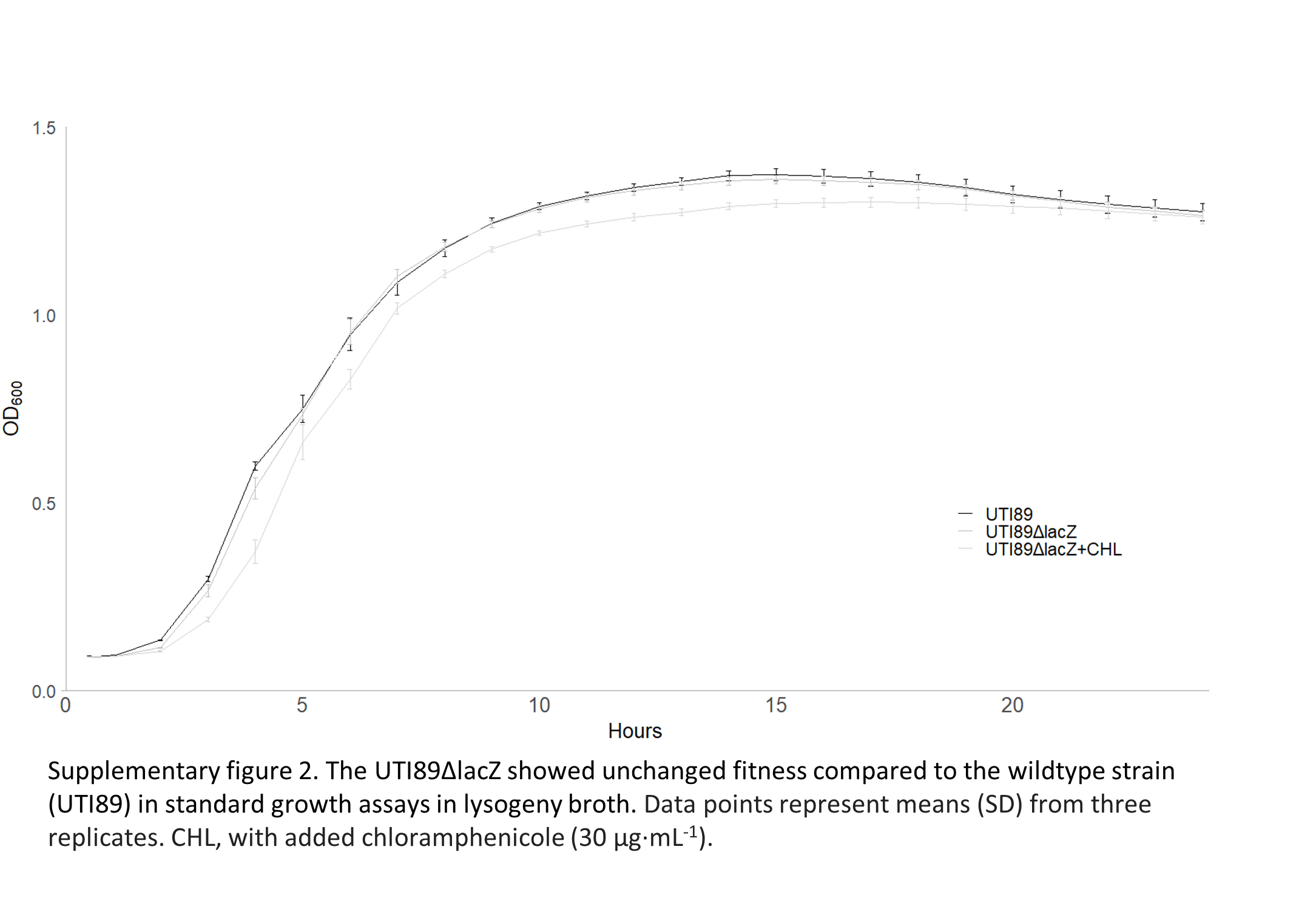

Supplement: jiae404_Supplementary_Data [file jiae404_Supplementary_Data.zip › Supplementary_figure2.png]
